# Use of clinical tolerance limits for assessing agreement

**DOI:** 10.1177/09622802221137743

**Published:** 2022-11-09

**Authors:** Patrick Taffé

**Affiliations:** Center for Primary Care and Public Health (unisanté), Division of Biostatistics, 27213 University of Lausanne, Lausanne, Switzerland

**Keywords:** Agreement, limits of agreement, tolerance limits, differential bias, proportional bias, method comparison

## Abstract

In this study, we have further extended the methodology proposed, first, by Lin et al. (2002) and, later, extended by Stevens et al. (2017, 2018), on the coverage probability/probability of agreement, by relaxing the strong parametric assumptions regarding the distribution of the latent trait and developing inference methods allowing to compute both pointwise and simultaneous confidence bands. The methodology requires repeated measurements by at least one of the two measurement methods and accommodates heteroscedastic measurement errors. It performs often very well even when one has only one measurement by one of the two measurement methods and at least five repeated measurements from the other. It circumvents some of the deficiencies of the Bland & Altman limits of agreement method and provides a more direct assessment of the agreement level.

## 1 Introduction

In clinical research, to assess the agreement/interchangeability between two measurement methods, when the characteristic of interest is continuous, the Bland & Altman's limits of agreement (LoA) method^1,2^ is one of the most frequently used methodologies (their 1986^2^ paper has been cited over 50,000 times as of May 2022). Often this is motivated by a new and perhaps less expensive or easier method of measurement against an established reference standard. To evaluate the comparability of the methods, the investigator collects repeated measurements from each method on a sample of subjects. Bland & Altman's LoA are then computed by adding and subtracting 1.96 times the estimated standard deviation from the mean differences, that is, the average bias estimate. A scatter plot of the differences versus the means of the two variables with the LoA superimposed is used to visually appraise the level of agreement. Further, a regression of the differences as a function of the means is added to the plot to indicate the direction and amplitude of the bias.^3^ A final decision regarding the agreement between the two methods is based on whether the LoA is within pre-defined clinical tolerance limits.

However, it has been shown that in the presence of a proportional bias or situations where the variances of the measurement errors of each of the methods are not constant (i.e. heteroscedastic) the Bland & Altman's plot may be misleading.^4^ When this is the case, the regression line may show an upward or downward trend when there is no bias or a zero slope when there is a bias. Therefore, recently, a new statistical methodology to assess the bias, precision, and agreement of a new measurement method, which circumvents the deficiencies of the Bland & Altman's LoA method, has been developed.^4,5^ This methodology, however, is in the same spirit as that of Bland & Altman, and the level of agreement is not directly quantified (except for the concept of “percentage of agreement,” which, however, does not depend on pre-defined tolerance limits). Rather, based on inspection of the Bias, Precision, and Agreement plots, the investigator has to decide whether disagreement (not agreement) is not too high for the two methods to be deemed to be interchangeable.

To more directly quantify the level of agreement, Lin et al.^6^ have proposed the concept of “coverage probability,” where the probability that the absolute difference between the two measurements made on the same subject is less than a pre-defined value is computed. Their methodology, however, allows one to assess the overall agreement and does not take into account that the level of agreement might depend on the value of the true latent trait in a continuous way. In addition, implicitly, homoscedastic measurement errors are assumed (an often too strong assumption) and the presence of a possible bias is not assessed. For these reasons, Stevens et al.^7^ have extended this methodology to allow the coverage probability to depend on the value of the latent trait, as well as on the amount of bias. Later, they further extended their methodology to allow for heteroscedastic measurement errors.^8^ They have called their extended agreement concept “probability of agreement.”

There are, however, several important limitations to the Stevens et al.^7,8^ methodology. First of all, it essentially relies on a parametric specification of the distribution of the latent trait, which is unpleasant as usually little is known about this distribution, particularly given that measurement errors make its identification difficult. In addition, the investigator has to decide over which support the index will be computed, thereby possibly extrapolating to impossible or unrealistic values of the genuine latent trait. Third, they considered constant tolerance limits, whereas often the variance of the measurement errors is heteroscedastic and increases with the value of the latent trait. Consequently, non-constant tolerance limits, which depend on the latent trait might be better suited. Fourth, they computed pointwise, and not simultaneous, confidence bands around the probability of agreement, which bears important limitations, as in practice one may be interested in assessing the agreement level at different values of the latent trait or even across an entire interval and not just at a single point of the support. Fifth, they did not show any simulation results regarding the coverage rate of their confidence band, nor used, for example, the logit transformation to avoid negative values to be included in the confidence band (as illustrated by Figure 2 in their 2018 paper^7^). Actually, their developments were based on the use of the delta method, which may not always provide reliable results (as demonstrated by the results of the present study), particularly when the function of the parameters is highly non-linear. Indeed, the same issue with the delta method has been demonstrated in a recently published study.^5^ Sixth, since the probability of agreement index depends on the value of the true trait, which is by definition latent and not estimated in their approach, it is practically not possible to assess the true level of agreement. Lastly, their unconditional probability of agreement concept,^7^ to assess the overall agreement level, is not a proper unconditional or marginal probability; rather it is a conditional probability evaluated at the mean value of the latent trait and therefore it does not summarize the overall agreement appropriately.

Many other methods to assess agreement/interchangeability have been proposed in the literature and we refer the interested reader to some recently published papers for a more extensive literature review.^8–10^

Therefore, in this paper, the main goal is to develop an extended methodology, based on Lin et al.^6^ and Steven et al.^8^ coverage probability/probability of agreement concepts, which overcomes the above-mentioned limitations. For this, we will rely on an empirical Bayes approach (to predict the value of the true latent trait), which has already been fruitfully used to overcome the limitations of Bland & Altman's LoA methodology.^4,5^ The inference will be developed to build both pointwise and simultaneous confidence bands around the conditional agreement curve, such that the investigator may adopt either method according to his inference goal.

## 2 Methodology of the clinical tolerance limits

Consider the general measurement error model:

(1) $$y_{1ij}=\alpha_{1}+\beta_{1}x_{ij}+\varepsilon_{1ij},\;\varepsilon_{1ij}|x_{ij}∼N(0,\sigma_{\varepsilon_{1}}^{2}(x_{ij};\theta_{1}))$$

$$y_{2ij}=\alpha_{2}+\beta_{2}x_{ij}+\varepsilon_{2ij},\;\varepsilon_{2ij}|x_{ij}∼N(0,\sigma_{\varepsilon_{2}}^{2}(x_{ij};\theta_{2}))$$

$$x_{ij}∼f_{x}(\mu_{x},\sigma_{x}^{2})$$

where $y_{1ij}$ be the *j*th replicate measurement by method 1 on individual *i*, $j=1,\ldots ,n_{i}$ and i=1,…,N, whereas $y_{2ij}$ is obtained by method 2, $x_{ij}$ is a latent variable with density $f_{x}$ representing the true unknown trait, and $\varepsilon_{1ij}$ and $\varepsilon_{2ij}$ represent measurement errors by methods 1 and 2. It is assumed that the variances of these errors, that is, $\sigma_{\varepsilon_{1}}^{2}(x_{ij};\theta_{1})$ and $\sigma_{\varepsilon_{2}}^{2}(x_{ij};\theta_{2})$, are heteroscedastic and increase with the level of the true latent trait $x_{ij}$ in a way to be precisely specified later, which depends on the vectors of unknown parameters $\theta_{1}$ and $\theta_{2}$. For the reference method, for instance, method 2, $\alpha_{2}=0$ and $\beta_{2}=1$, whereas for method 1, the differential $\alpha_{1}$ and proportional $\beta_{1}$ biases have to be estimated from the data. The mean value of the latent variable $x_{ij}$ is $\mu_{x}$ and its variance $\sigma_{x}^{2}$. It is assumed that the latent variable represents the true unknown but constant value of the trait for individual *i* and, therefore, $x_{ij}\equiv x_{i}$ (this assumption may be relaxed to allow, e.g. a linear trend^4^).

When method 2 is the reference standard and method 1 is the new method to be evaluated, the model reduces to:

(2) $$y_{1ij}=\alpha_{1}+\beta_{1}x_{i}+\varepsilon_{1ij},\;\varepsilon_{1ij}|x_{i}∼N(0,\sigma_{\varepsilon_{1}}^{2}(x_{i};\theta_{1}))$$

$$y_{2ij}=x_{i}+\varepsilon_{2ij},\;\varepsilon_{2ij}|x_{i}∼N(0,\sigma_{\varepsilon_{2}}^{2}(x_{i};\theta_{2}))$$

$$x_{i}∼f_{x}(\mu_{x},\sigma_{x}^{2})$$

Note that this measurement error model is slightly different from the classical measurement error model^11^ in that the heteroscedasticity depends on the latent trait and not on an observed average. In addition, we have considered a simple linear relationship between $y_{1ij}$ and $x_{i}$ to identify the differential and proportional biases. It is possible, however, to consider instead a non-linear function of $x_{i}$ but in that case, the bias no longer decomposes into two components with nice interpretations.

### 2.1 Computation of the conditional and overall/marginal agreement

Consider the differences:

(3) $$d_{ij}=y_{1ij}-y_{2ij}=(\alpha_{1}+\beta_{1}x_{i}+\varepsilon_{1ij})-(x_{i}+\varepsilon_{2ij})=\alpha_{1}+(\beta_{1}-1)x_{i}+(\varepsilon_{1ij}-\varepsilon_{2ij})$$

and assume that lower $C_{L}(x_{i})$ and upper $C_{U}(x_{i})$ clinical tolerance limits, which may depend on the true trait, have been defined a priori, that is, before seeing the data, based on clinical considerations, such that the Bernoulli variable $X_{ij}$ will take value 1 when the difference $d_{ij}$ lies between the two clinical tolerance limits and 0 otherwise:

$$X_{ij}=\left\{ \begin{array}{c} 1\;\;\;\mathrm{if}\;\;\;C_{L}(x_{i})<d_{ij}<C_{U}(x_{i})\\ 0\;\;\;\mathrm{otherwise} \end{array}\right.$$

We have $X_{ij}|x_{i}∼Bernoulli(\pi (x_{i}))$, where $\pi (x_{i})$ is the *coverage probability*^6^ or *conditional probability of agreement*.^7,8^

One may obtain the *overall/marginal agreement* by marginalizing:

(4) $$P(C_{L}(x_{i})<d_{ij}<C_{U}(x_{i}))\equiv \pi =\int_{-\infty }^{\infty }\pi (x_{i})f_{X}(x_{i})dx$$

Given model (2) and assumptions, one may compute the *conditional probability of agreement*:

(5) $$\begin{array}{rl} P(C_{L}(x_{i})<d_{ij}<C_{U}(x_{i})|x_{i})\equiv \pi (x_{i})\\ & =P\left(\frac{C_{L}(x_{i})-\alpha_{1}-(\beta_{1}-1)x_{i}}{\sqrt{V(\varepsilon_{1ij}-\varepsilon_{2ij})}}<\frac{d_{i}-\alpha_{1}-(\beta_{1}-1)x_{i}}{\sqrt{V(\varepsilon_{1ij}-\varepsilon_{2ij})}}<\frac{C_{U}(x_{i})-\alpha_{1}-(\beta_{1}-1)x_{i}}{\sqrt{V(\varepsilon_{1ij}-\varepsilon_{2ij})}}|x_{i}\right)\\ & =\Phi \left(\frac{C_{U}(x_{i})-\alpha_{1}-(\beta_{1}-1)x_{i}}{\sqrt{\sigma_{\varepsilon_{1}}^{2}(x_{i};\theta_{1})+\sigma_{\varepsilon_{2}}^{2}(x_{i};\theta_{2})}}\right)-\Phi \left(\frac{C_{L}(x_{i})-\alpha_{1}-(\beta_{1}-1)x_{i}}{\sqrt{\sigma_{\varepsilon_{1}}^{2}(x_{i};\theta_{1})+\sigma_{\varepsilon_{2}}^{2}(x_{i};\theta_{2})}}\right) \end{array}$$

where Φ is the standard normal cumulative distribution function.

As regards the clinical tolerance limits, $C_{L}(x_{i})$ and $C_{U}(x_{i})$, it is up to the investigator to define them, ideally based on clinical considerations, before seeing the data.

One simple alternative is to set constant values, which do not depend on the true latent trait:

$$C_{L}(x_{i})=a$$

$$C_{U}(x_{i})=b$$

Alternatively, one may define limits that depend on the true latent trait in a specific form, for example, linear:

$$C_{L}(x_{i})=-a-bx_{i}$$

$$C_{U}(x_{i})=a+bx_{i}$$

where “*a*” represents the smallest tolerable upper limit for a zero value of the latent trait and “*b*” is the percentage of acceptable difference beyond the zero latent trait value.

Other choices are of course possible and the tolerance limits need not be linear.

### 2.2 Estimation of the model parameters

We do not want to make strong distributional assumptions regarding the true latent trait $x_{i}$ and instead of specifying a parametric distribution, and treating $x_{i}$ as a nuisance parameter to be integrated out from the joint likelihood function,^12^ we estimate the regression model for $y_{2ij}$ by marginal maximum likelihood (i.e. without specifying any parametric distribution for $x_{i}$) and adopt an empirical Bayes approach^13^ to predict $x_{i}$, by means of its posterior distribution (i.e. the mean of the conditional distribution of $x_{i}$ given the vector $y_{2i}$, which is the best linear unbiased prediction (BLUP) for $x_{i}$):

(6) $${\hat{x}}_{i}=E(x_{i}|y_{2i})$$

(7) $$=\int x_{i}\frac{\;f_{y_{2}}(y_{2i}|x_{i})f_{x}(x_{i})}{\int \;f_{y_{2}}(y_{2i}|x_{i})f_{x}(x_{i})\;dx_{i}}dx_{i}$$

where for the sake of notational convenience we have suppressed the dependence of the density functions $f_{y_{2}}$ and $f_{x}$ from their parameters, which have been estimated by maximum likelihood.^4^

Notice, that with sufficient repeated observations per individual (e.g. between 5 and 10) and a sufficient number of individuals (e.g. 100) the empirical distribution of the BLUP of $x_{i}$ will approximate well the true distribution, as can be verified empirically by simulations and shown theoretically.^14^ Then, one proceeds to the estimation of the regression equation for $y_{1ij}$ in (2) and of the differential $\alpha_{1}$ and proportional $\beta_{1}$ biases simply by OLS after having substituted the BLUP ${\hat{x}}_{i}$ for the true unmeasured trait $x_{i}$.^4^

Estimates of the standard deviation of the measurement errors are obtained following a similar approach to that of Bland and Altman^3,4^:

(8) $${\hat{\sigma }}_{\varepsilon 1}({\hat{x}}_{i};{\hat{\theta }}_{1})\;=\hat{E}(\;|{\hat{\varepsilon }}_{1ij}^{*}|\;)\sqrt{\pi /2}=({\hat{\theta }}_{1}^{\left(0\right)}+{\hat{\theta }}_{1}^{\left(1\right)}{\hat{x}}_{i})\sqrt{\pi /2}$$

(9) $${\hat{\sigma }}_{\varepsilon_{2}}({\hat{x}}_{i};{\hat{\theta }}_{2})\;=\hat{E}(\;|{\hat{\varepsilon }}_{2ij}^{*}|\;)\sqrt{\pi /2}=({\hat{\theta }}_{2}^{\left(0\right)}+{\hat{\theta }}_{2}^{\left(1\right)}{\hat{x}}_{i})\sqrt{\pi /2}$$

Notice, that the form of the heterogeneity needs not to be a straight line and a fractional polynomial may be used instead if the investigator believes that the straight-line model is too restrictive. In any case, a graphical representation of $|{\hat{\varepsilon }}_{2ij}^{*}|\;$ versus ${\hat{x}}_{i}$ may be useful to visually check the plausibility of the straight-line model.

Once the parameters have been estimated and conditional agreement computed:

(10) $$\hat{\pi }({\hat{x}}_{i})=\Phi \left(\frac{C_{U}({\hat{x}}_{i})-{\hat{\alpha }}_{1}-({\hat{\beta }}_{1}-1){\hat{x}}_{i}}{\sqrt{\sigma_{\varepsilon_{1}}^{2}({\hat{x}}_{i};{\hat{\theta }}_{1})+\sigma_{\varepsilon_{2}}^{2}({\hat{x}}_{i};{\hat{\theta }}_{2})}}\right)-\Phi \left(\frac{C_{L}({\hat{x}}_{i})-{\hat{\alpha }}_{1}-({\hat{\beta }}_{}-1){\hat{x}}_{i}}{\sqrt{\sigma_{\varepsilon_{1}}^{2}({\hat{x}}_{i};{\hat{\theta }}_{1})+\sigma_{\varepsilon_{2}}^{2}({\hat{x}}_{i};{\hat{\theta }}_{2})}}\right)$$

the variance needs to be computed to build pointwise and simultaneous confidence bands.

To proceed, we will rely on two different approaches, the first, the conventional multivariate delta method, and the second, based on a simulation method. In our experience, with complicated functions involving ratios of random variables, the delta method may perform poorly.^5^ Also, to guarantee confidence bands comprising values between 0 and 1, the logit transform (of the conditional agreement) will be used.

When the goal is to assess the agreement level for a specific value of the latent trait, a pointwise confidence interval will be fine as it guarantees that on average 95% of the computed intervals will cover the true value. However, when interest lies in several points from the support or in the whole curve, a simultaneous confidence band is required, as it guarantees a proper coverage rate for the simultaneous inference, whatever the number of points from the support.

### 2.3 Computation of pointwise and simultaneous confidence bands

The variance of $\hat{\pi }({\hat{x}}_{i})$ may be computed by using the multivariate delta method and the uncertainty in the estimate ${\hat{x}}_{i}$ accounted for by the law of total variance.^5^ However, as mentioned above and based on previous experience,^5^ the performance of the delta method may not always be good and one needs to consider alternative methods to compute the variance. One way of doing this is by simulations:

- Step 1: For each individual *i*, 1000 values for ${\hat{x}}_{i}$ are drawn in $N({\hat{x}}_{i},V({\hat{x}}_{i}-x_{i}))$.
- Step 2: As ${\hat{\theta }}_{k}^{\left(l\right)}\overset{asy}{∼}\;N({\hat{\theta }}_{k}^{\left(l\right)},V({\hat{\theta }}_{k}^{\left(l\right)})),\;\;k=1,2,\;\;l=0,1,$ 1000 values for ${\hat{\theta }}_{1}^{\left(0\right)}$ and ${\hat{\theta }}_{1}^{\left(1\right)}$, and for ${\hat{\theta }}_{2}^{\left(0\right)}$ and ${\hat{\theta }}_{2}^{\left(1\right)}$, are drawn in a bivariate Normal distribution, accounting for the $\mathrm{cov}({\hat{\theta }}_{k}^{\left(0\right)};{\hat{\theta }}_{k}^{\left(1\right)})$.
- Step 3: 1000 values for ${\hat{\alpha }}_{1}$ and ${\hat{\beta }}_{1}$ are drawn in a bivariate Normal distribution, accounting for the $\mathrm{cov}({\hat{\alpha }}_{1};{\hat{\beta }}_{1})$.
- Step 4: Finally, 1000 values for $\hat{\pi }({\hat{x}}_{i})$ are generated for each individual *i* and the sample variance computed.

A pointwise 95% confidence band for $\pi (x_{i})$ may be computed either directly, by referring to the 2.5 and 97.5 percentiles of the simulated distribution, or indirectly, by using the simulated variance and Normal approximation on the logit scale:

(11) $$\begin{array}{r} \mathrm{logit}(\hat{\pi }({\hat{x}}_{i}))-Z_{1-\alpha /2}\sqrt{V(\mathrm{logit}(\hat{\pi }({\hat{x}}_{i})))}\leq \mathrm{logit}(\pi (x_{i}))\\ \;\leq \mathrm{logit}(\hat{\pi }({\hat{x}}_{i}))+Z_{1-\alpha /2}\sqrt{V(\mathrm{logit}(\hat{\pi }({\hat{x}}_{i})))} \end{array}$$

where $Z_{1-\alpha /2}$ is either the quantile of the standard Normal distribution or Student distribution with N−6 degrees of freedom (i.e. number of individuals minus number of parameters estimated).

With regard to a 100(1−α) % *simultaneous* confidence band, the following inequality must hold^15,16^:

(12) $$P\left(\pi (x_{i})\in \left[\hat{\pi }({\hat{x}}_{i})\pm q_{1-\alpha }\sqrt{V(\hat{\pi }({\hat{x}}_{i})-\pi (x_{i}))}\right],\;\forall x_{i}\in \Psi \right)\geq 1-\alpha$$

where Ψ denotes the set of $x_{i}$ values of interest and $q_{1-\alpha }$ a critical value to be determined.

The probability statement (12) is equivalent to:

(13) $$P\left(\underset{\min\leq x_{i}\leq \max}{\sup}\frac{\left|\hat{\pi }({\hat{x}}_{i})-\pi (x_{i})\right|}{\sqrt{V(\hat{\pi }({\hat{x}}_{i})-\pi (x_{i}))}}\leq q_{1-\alpha }\right)\geq 1-\alpha$$

where min is the minimum and max maximum value of $x_{i}$. The appropriate value of $q_{1-\alpha }$ can be found by simulating the left-hand side quantity in the probability statement (13), say a thousand times, and computing the 1−α empirical quantile of the distribution.

As in our case, as $x_{i}$ is a latent trait and not observed, $q_{1-\alpha }$ is computed as the 1−α quantile of the sampling distribution of^5^:

(14) $$\underset{\min\leq {\hat{x}}_{i}\leq \max}{\sup}\frac{\left|{\hat{\pi }}^{\left(m\right)}({\hat{x}}_{i})-\hat{\pi }({\hat{x}}_{i})\right|}{\sqrt{V({\hat{\pi }}^{\left(m\right)}({\hat{x}}_{i}))}}$$

where ${\hat{\pi }}^{\left(m\right)}({\hat{x}}_{i})$ represents the *m*th simulated value of $\hat{\pi }({\hat{x}}_{i})$, m=1,…,R, and $\hat{\pi }({\hat{x}}_{i})$ is the estimated value of $\pi (x_{i})$ based on the sample at hand. The number *R* of simulations should be large enough (e.g. 1000) to get stable estimates.

Concretely, a 100(1−α) % simultaneous confidence band for the conditional agreement is given by:

(15) $$\hat{\pi }({\hat{x}}_{i})-q_{1-\alpha }\sqrt{V(\hat{\pi }({\hat{x}}_{i}))}\leq \pi (x_{i})\leq \hat{\pi }({\hat{x}}_{i})+q_{1-\alpha }\sqrt{V(\hat{\pi }({\hat{x}}_{i}))}$$

where $q_{1-\alpha }$ is found by using the following simulation algorithm:

- Step 1: ${\hat{\alpha }}_{1}^{\left(m\right)}$ and ${\hat{\beta }}_{1}^{\left(m\right)}$ are simulated from the appropriate bivariate Normal distribution and the ${\hat{x}}_{i}^{\left(m\right)}$ are drawn in $N({\hat{x}}_{i},V({\hat{x}}_{i}-x_{i}))$. Then, ${\hat{\pi }}^{\left(m\right)}({\hat{x}}_{i}^{\left(m\right)})$ and $V({\hat{\pi }}^{\left(m\right)}({\hat{x}}_{i}^{\left(m\right)}))$ are computed for each simulation *m*.
- Step 2: For m=1,…,R calculate:
  $$q_{1-\alpha }^{\left(m\right)}=\underset{\min\leq {\hat{x}}_{i}\leq \max}{\sup}\;\left|\frac{{\hat{\pi }}^{\left(m\right)}({\hat{x}}_{i}^{\left(m\right)})-\hat{\pi }({\hat{x}}_{i})}{\sqrt{V({\hat{\pi }}^{\left(m\right)}({\hat{x}}_{i}^{\left(m\right)}))}}\right|$$
- Step 3: $q_{1-\alpha }$ is the 1−α quantile of the empirical distribution of the $q_{1-\alpha }^{\left(m\right)}$.

To guarantee values comprised strictly between 0 and 1, the logit transform (of the conditional agreement) is used in all the calculations and the inverse transform is applied in the end to get the required band.

The performance of these alternative variance estimators and confidence bands has been studied in the simulation section below.

## 3 A simulation study

The goal of this simulation study is to assess the performance of the various confidence bands developed in section 2 and introduce the “Conditional agreement plot.”

### 3.1 Performance of the confidence bands

We simulated 1000 data sets according to the following data generating process:

(16) $$\begin{array}{r} y_{1ij}=\alpha_{1}+\beta_{1}x_{i}+\varepsilon_{1ij},\;\varepsilon_{1ij}|x_{i}∼N(0,(\theta_{1}^{\left(0\right)}+\theta_{1}^{\left(1\right)}x_{i}{)}^{2}\pi /2)\\ y_{2ij}=x_{i}+\varepsilon_{2ij},\;\;\;\;\;\;\;\;\;\;\;\;\;\;\;\;\;\varepsilon_{2ij}|x_{i}∼N(0,(\theta_{2}^{\left(0\right)}+\theta_{2}^{\left(1\right)}x_{i}{)}^{2}\pi /2) \end{array}$$

$$x_{i}∼U\;[a,\;b]$$

where i=,1,…,N individuals, the number of repeated measurements $n_{2i}∼U\;\{c,d\}$ by the reference method was drawn in a discrete Uniform distribution, and for the new measurement method $n_{1i}∼U\;\{e,f\}$, and by varying the values of the vector of parameters $[\alpha_{1}\;\beta_{1}\;\theta_{1}^{\left(0\right)}\;\theta_{1}^{\left(1\right)}\;\theta_{2}^{\left(0\right)}\;\theta_{2}^{\left(1\right)}{]}^{\prime }$.

For the sake of brevity, we focused on five different settings, which may be relevant for the clinical practice: (1) zero differential and no proportional biases; (2) negative differential bias with proportional bias less than 1; (3) negative differential bias with proportional bias larger than 1; (4) positive differential bias with proportional bias less than 1; (5) positive differential bias with proportional bias larger than 1. To allow comparisons with results from a previous study,^5^ the following pairs of values for the differential and proportional biases were used $(\alpha_{1},\beta_{1})=$(0, 1), (−6, 0.85), (−4, 1.2), (4, 0.8), (4, 1.2), and the level of the latent trait assumed to be uniformly distributed between 10 and 40. Other distributions for $x_{i}$ have also been investigated (Normal and Gamma).

The number of repeated measurements from the reference standard was drawn in the three distributions: (1) $n_{2i}∼U\{10,15\}$; (2) $n_{2i}∼U\{5,10\}$; (3) $n_{2i}∼U\;\{1,3\}$, and that from the new measurement method in the two distributions: (1) $n_{1i}∼U\;\{1,3\}$; (2) $n_{1i}=1$. Regarding the number of individuals, the distribution of the latent trait, and the parameters of the variance functions, we restricted the presentation to the results obtained with n=100, $x_{i}∼U\;[10,40]$, and for some selected values of ( $\theta_{1}^{\left(0\right)}$, $\theta_{1}^{\left(1\right)}$, $\theta_{2}^{\left(0\right)}$, $\theta_{2}^{\left(1\right)}$): (0, 0.2, 1.75, 0.08), (0.1, 0.07, 0.15, 0.09), and (1, 0.05, 2, 0.01) (these values and those for the differential and proportional biases were selected to illustrate settings where Bland & Altman's methodology provided strongly biased estimates of the differential and proportional biases).

Notice that $n_{2i}∼U\;\{1,3\}$ means on average two repeated measurements by the reference standard, which is an extremely unfavorable scenario given that the methodology requires several measurements per individual by at least one of the two measurement methods. Simulation results and conclusions for various sample sizes and levels of heteroscedasticity were similar.

As regards the clinical tolerance limits, for the sake of simplicity, they have been set to constant values and do not depend on the true latent trait:

$$C_{L}(x_{i})=-5$$

$$C_{U}(x_{i})=5$$

Results of the simulations regarding coverage rates (nominal set at 95%) of the pointwise and simultaneous confidence bands are presented in Tables 1 and 2. In Table 1, the cells contain coverage rates obtained by the Normal approximation, quantiles of the simulation distribution, and delta method (separated by slashes).

**Table 1.** Coverage rates (nominal 95%) of the pointwise confidence band (Normal approximation/quantiles of the simulation distribution/delta method).

Differential bias	Proportional bias	$n_{2i}∼U\;\{10,15\}$	$n_{2i}∼U\;\{5,10\}$	$n_{2i}∼U\;\{1,3\}$	$\theta_{1}^{\left(0\right)},\theta_{1}^{\left(1\right)},\theta_{2}^{\left(0\right)},\theta_{2}^{\left(1\right)}$
$n_{1i}∼U\;\{1,3\}$					
0	1	94.6/94.7/88.0	94.7/94.9/88.2	92.6/93.2/84.9	0, 0.2, 1.75, 0.08
−6	0.85	94.8/93.8/90.5	93.7/92.2/89.0	87.9/83.3/81.7	0.1, 0.07, 0.15, 0.09
−4	1.2	94.1/94.0/90.2	93.5/93.5/89.7	90.1/91.0/86.4	1, 0.05, 2, 0.01
4	0.8	94.9/94.7/89.7	94.6/94.4/89.1	93.6/92.7/87.6	0, 0.2, 1.75, 0.08
4	1.2	94.6/93.7/94.0	95.8/94.9/95.3	96.1/95.1/95.1	0, 0.2, 1.75, 0.08
$n_{1i}=1$					
0	1	94.6/94.7/88.2	94.1/94.3/87.4	91.7/92.6/83.4	0, 0.2, 1.75, 0.08
−6	0.85	94.9/93.6/90.1	95.3/93.8/91.6	92.0/86.2/85.7	0.1, 0.07, 0.15, 0.09
−4	1.2	94.4/94.0/90.5	94.8/94.8/91.2	91.4/91.8/86.9	1, 0.05, 2, 0.01
4	0.8	95.3/95.0/90.2	94.8/94.6/89.7	93.8/93.1/87.8	0, 0.2, 1.75, 0.08
4	1.2	95.2/94.1/94.2	96.7/95.3/95.8	97.3/96.3/96.2	0, 0.2, 1.75, 0.08

**Table 2.** Coverage rates (nominal 95%) of the simultaneous confidence band.

Differential bias	Proportional bias	$n_{2i}∼U\;\{10,15\}$	$n_{2i}∼U\;\{5,10\}$	$n_{2i}∼U\;\{1,3\}$	$\theta_{1}^{\left(0\right)},\theta_{1}^{\left(1\right)},\theta_{2}^{\left(0\right)},\theta_{2}^{\left(1\right)}$
$n_{1i}∼U\;\{1,3\}$					
0	1	98.3	97.3	91.8	0, 0.2, 1.75, 0.08
−6	0.85	99.4	99.1	98.4	0.1, 0.07, 0.15, 0.09
−4	1.2	98.9	98.5	94.4	1, 0.05, 2, 0.01
4	0.8	98.3	97.6	94.4	0, 0.2, 1.75, 0.08
4	1.2	99.5	99.5	97.0	0, 0.2, 1.75, 0.08
$n_{1i}=1$					
0	1	99.5	97.5	91.2	0, 0.2, 1.75, 0.08
−6	0.85	99.7	99.2	99.6	0.1, 0.07, 0.15, 0.09
−4	1.2	99.6	99.1	97.4	1, 0.05, 2, 0.01
4	0.8	99.7	97.7	96.0	0, 0.2, 1.75, 0.08
4	1.2	99.6	99.5	98.4	0, 0.2, 1.75, 0.08

Coverage rates are generally very good when the variance has been computed by the simulation method and the Normal approximation is used, and at least five to 10 repeated measurements per individual are available by the reference method. With few repeated measurements per individual, the coverage rate of the method based on the quantiles of the simulation distribution has not performed as well as that based on the simulated variance and Normal approximation. Globally, the coverage rate of the delta method is not good.

For example, when $n_{1i}=1$, $n_{2i}∼U\{10,15\}$, differential bias is 0 and proportional bias 1, and the clinical tolerance limits have been prespecified as $C_{L}(x_{i})=-5$ and $C_{U}(x_{i})=5$, the coverage rate of the pointwise 95% confidence band using the Normal approximation and quantiles of the simulation distribution is 94.6% and 94.7%, whereas it is only 88.2% for the delta method.

Coverage rates are generally on the conservative side with at least five to 10 repeated measurements per individual by the reference method.

### 3.2 The conditional agreement plot

To introduce the “Conditional agreement plot,” the following data generating process has been considered:

(17) $$y_{1i}=4+0.8x_{i}+\varepsilon_{1i},\;\varepsilon_{1i}|x_{i}∼N(0,(0.2x_{i}{)}^{2})$$

$$y_{2ij}=x_{i}+\varepsilon_{2ij},\;\varepsilon_{2ij}|x_{i}∼N(0,(1.75+0.08\;x_{i}{)}^{2})$$

$$x_{i}∼U\;[10,100]$$

where i=1,…,100 and the number of repeated measurements per individual *i* has been set to $n_{1i}=n_{2i}=5$. The new method (method 1) has a differential bias of 4 and a proportional bias of 0.8. In addition, the variance of the measurement errors from method 1 is larger than that of reference method 2.

Consider, first, the setting where the clinical tolerance limits have been set to constant values and do not depend on the true latent trait:

$$C_{L}(x_{i})=-5$$

$$C_{U}(x_{i})=5$$

**Figure 1.**
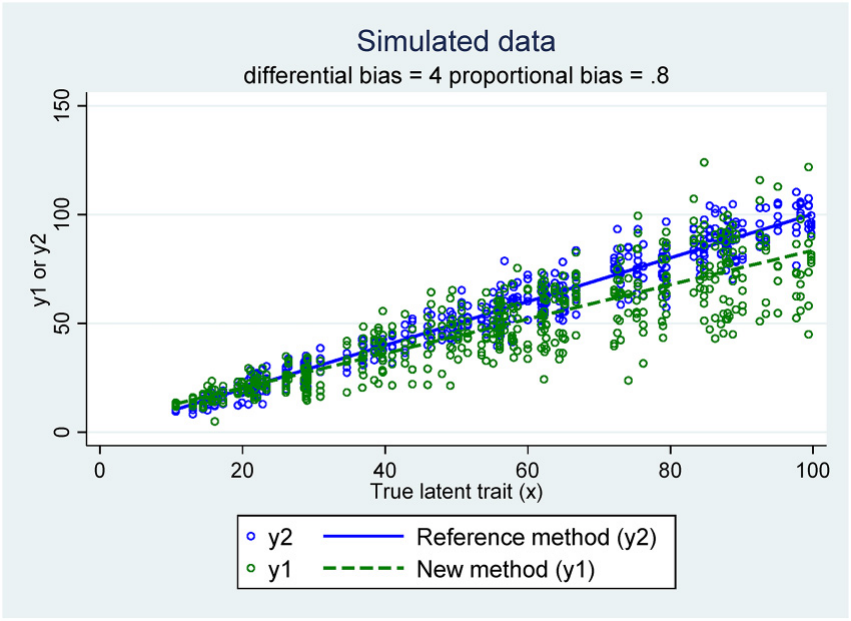
Scatter plot of the simulated data.

In Figure 1, a scatter plot of the simulated data versus the true latent trait is provided.

On the scatter plot of the simulated data the proportional bias of the new method is clearly apparent, as well as the heteroscedasticity of the measurement errors.

**Figure 2.**
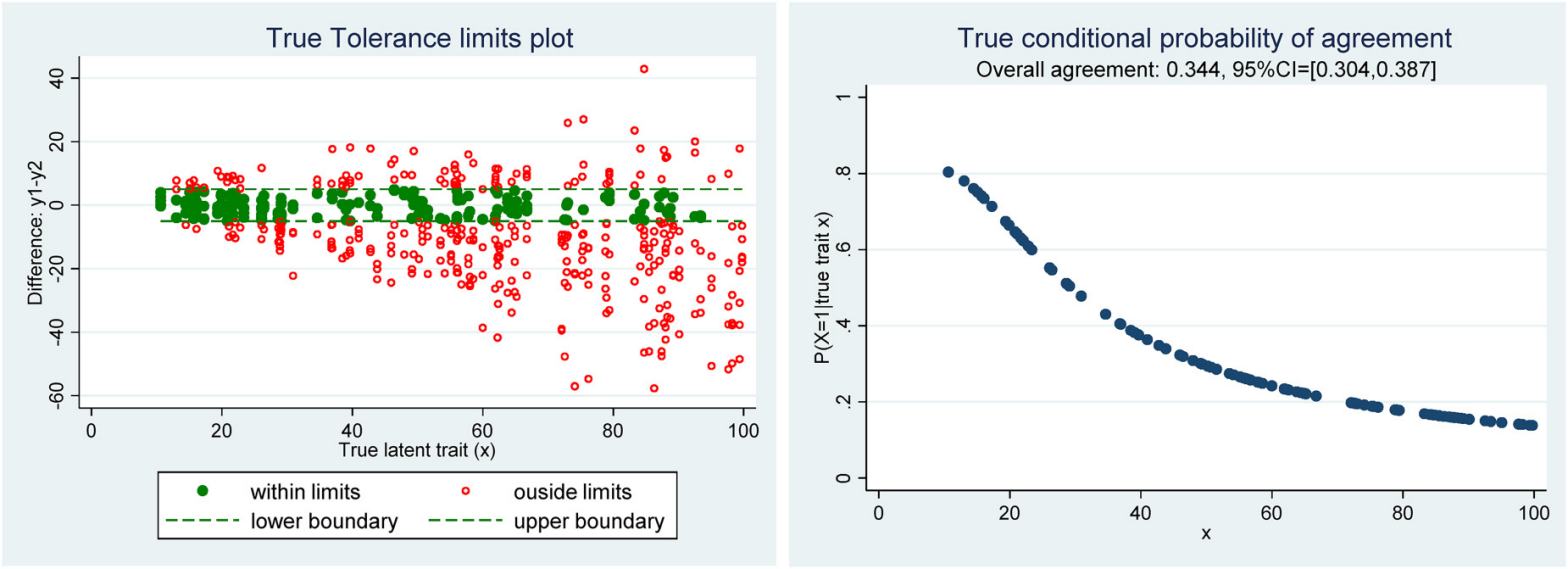
(Left) Scatter plot of the differences y_1_–y_2_ versus the true latent trait with tolerance limits, (right) scatter plot of the true conditional probability of agreement.

**Figure 3.**
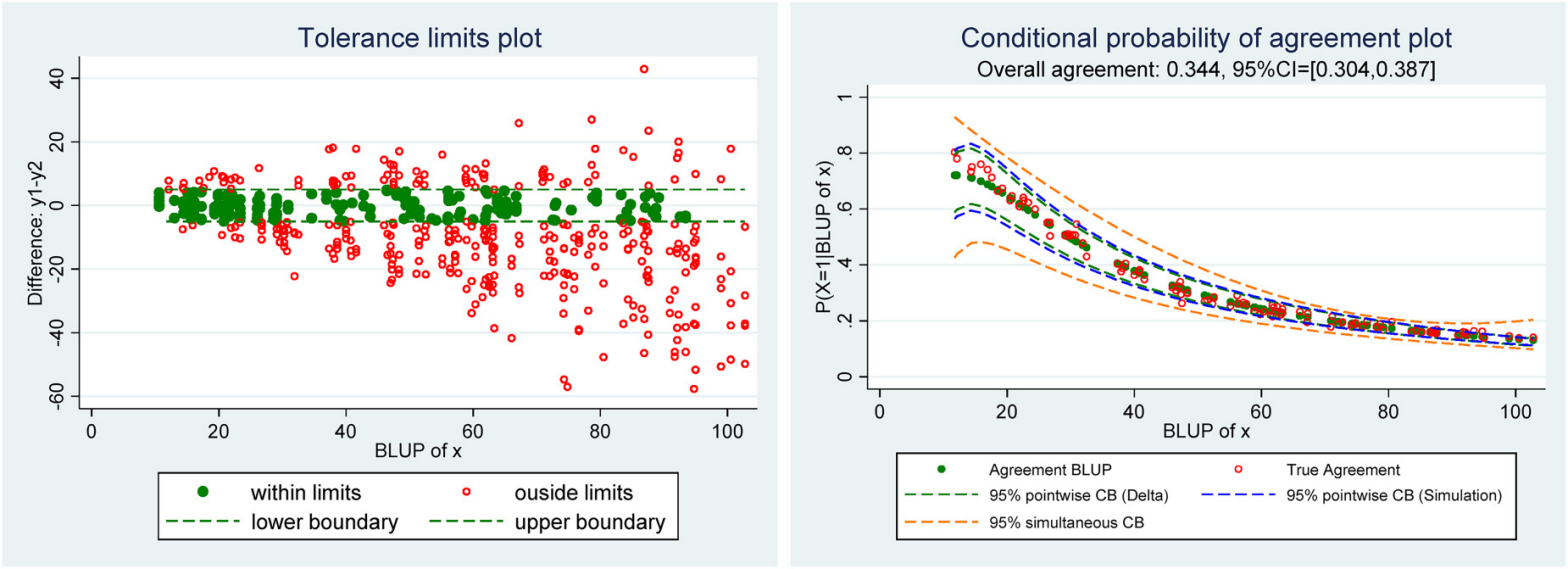
(Left) Scatter plot of the differences y_1_–y_2_ versus the BLUP of *x* with tolerance limits, (right) conditional probability of agreement plot.

Figure 2, left, shows the true Tolerance limit plot, which serves as a benchmark for the comparison with the Tolerance limit plot (Figure 3 left) in which the unknown true trait has been predicted by the empirical Bayes method (i.e. BLUP of $x_{i}$), and right the true conditional probability of agreement computed using formula (5).

In this example, due to the presence of a proportional bias and heteroscedasticity of measurement errors, the true conditional probability of agreement is not constant and decreases with the level of the true latent trait value ( $x_{i}$). The overall/marginal agreement has simply been estimated by the proportion of observations contained within the tolerance limits and the 95% confidence interval computed by the Agresti and Coull method.^17^

Based on the estimates of the parameters of model (2), the conditional probability of agreement and BLUP of the true latent trait $x_{i}$ have been computed. In Figure 3, we have drawn, left, the scatter plots of the differences versus the BLUP of $x_{i}$, called the “Tolerance limit plot,” and, right, the “Conditional probability of agreement plot.”

The Tolerance limits plot from Figure 3 is very similar to that of Figure 1, which illustrates that already with five repeated measurements per individual the BLUP of $x_{i}$ generally provides a very good approximation of the true latent trait.^4^ This plot may be useful to get a first glimpse of the agreement based on the predefined tolerance limits. On the Conditional agreement plot, one can see that the estimated (in green) and true values (in red) of the conditional agreement are pretty similar, except maybe for low values of the latent trait. We have added the 95% pointwise confidence bands (CB) computed by the simulation and delta methods. Simulation results in Table 1 have shown that the coverage rate of the simulation method was much better than that of the delta and the reason is well illustrated in this example where the pointwise CB calculated by the simulation method is wider than that computed by the delta method. As expected, the simultaneous CB is much wider than the pointwise and allows one to assess the probability of agreement for as many points from the support as desired.

**Figure 4.**
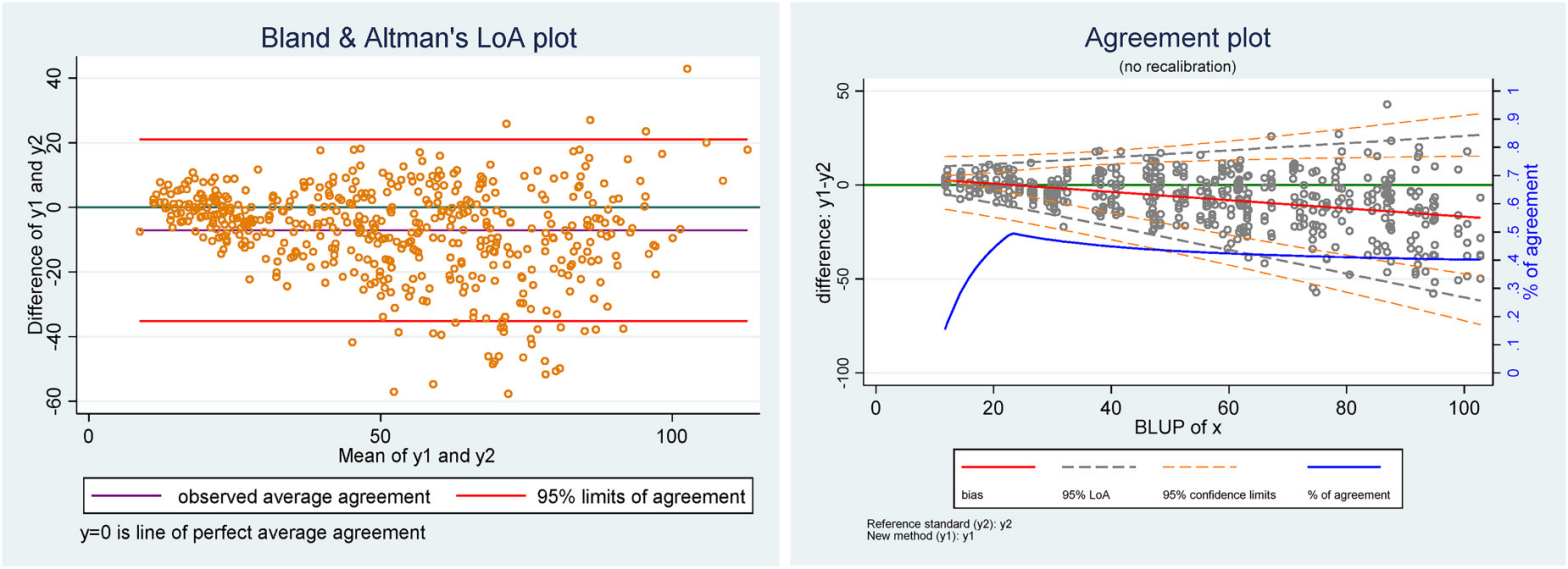
(Left) Bland & Altman's LoA plot, (right) agreement plot.

For the sake of comparison, in Figure 4 we have also computed the Bland & Altman's LoA and Taffé's agreement plots.

In comparison with the Conditional probability of agreement plot, which directly provides the level of agreement, the investigator must carefully inspect the LoA on the Bland & Altman's and Taffé's agreement plots to assess the degree of agreement. In this respect, the percentage of agreement (defined as $\text{\%}\hat{A}=1-(Z_{1-\alpha /2}{\hat{\sigma }}_{d}+|bias_{i}|\;)/{\hat{x}}_{i}$, which is roughly proportional to the half-width of the LoA plus the absolute amount of bias, see Taffé^5^), in the Agreement plot, may be helpful. Comparing the percentage of the agreement curve with the Conditional agreement plot, it is interesting to notice that for low values of the latent trait the two figures disagree: on the Agreement plot the level of agreement drops down for values of the latent trait below 25, whereas it is the highest on the Conditional agreement plot. Actually, this is not surprising as the percentage of agreement penalizes the width of the LoA by the absolute value of the bias, which is proportionally high for low values of the latent trait, whereas the tolerance limits have been set to constant values, irrespective of the latter.

To fix this issue, one may define tolerance limits that depend on the true latent trait in a specific form, for example, linearly:

$$C_{L}(x_{i})=-bx_{i}$$

$$C_{U}(x_{i})=bx_{i}$$

**Figure 5.**
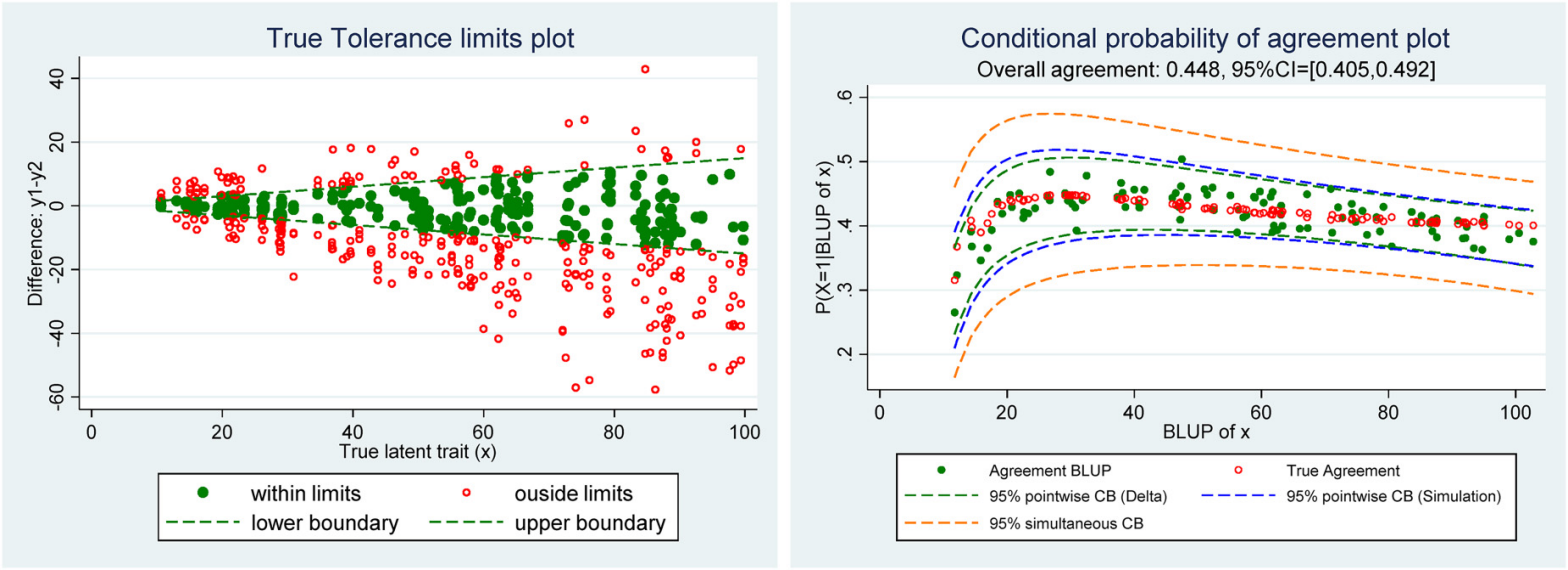
(Left) Scatter plot of the differences y_1_–y_2_ versus the BLUP of *x* with non-constant tolerance limits, (right) conditional probability of agreement plot.

To illustrate, in Figure 5 we have set $C_{L}(x_{i})=-0.15x_{i}$ and $C_{U}(x_{i})=0.15x_{i}$, such that the tolerance limits are narrower for small values and wider for large values of the latent trait.

Clearly, now, the percentage of the agreement curve and the Conditional agreement plot give the same message, the level of agreement is best for values of the latent trait around 25.

A worked example based on real data is presented in the Supplemental Appendix.

## 6 Discussion

In this study, we have further extended the methodology proposed, first, by Lin et al.^6^ and, then, extended by Stevens et al.,^8^ on the coverage probability/probability of agreement concepts, by relaxing the strong parametric assumptions regarding the distribution of the latent trait. In addition, we have extended the tolerance limits concept, which is allowed to depend on the true latent trait, and developed an inference theory based on different variance estimation methods. The focus has been on developing simultaneous, and not simply pointwise, confidence bands to allow for simultaneous inferences, that is, to allow the inference for the whole curve and not only for a specific value of the latent trait. This is particularly relevant from a clinical perspective, as it may turn out that the probability of agreement is high enough only for a limited range of the values of the latent trait. In addition, by superimposing on the same plot the conditional agreement curves obtained from several competing new measurement methods, along with their simultaneous confidence bands, one may assess which of these competing methods performs best and for which values of the latent trait.

We have investigated two different methods to compute the variance of the conditional agreement, the standard delta method, and a simulation method. Simulation results (in Table 1) have shown that the delta method has not performed well, whereas the simulation method performed very well when there were at least five to 10 repeated measurements per individual by the reference method and only one measurement by the new method. This poor result of the delta method has already been observed in Taffé^5^ and it is recommended to abandon it for the simulation method. It is worth mentioning that to avoid impossible negative values in the CB, the logit transform should be used when using the Normal approximation or delta method. Also, in some settings, the coverage rate of the pointwise confidence band based on the quantiles of the simulation distribution has not performed as well as that based on the simulated variance and Normal approximation, particularly with few repeated measurements per individual. The reason is not clear and should be investigated in further research.

The coverage rate of the simultaneous CB (in Table 2) has been found to be quite conservative in almost all the settings investigated. This result has already been observed in Taffé^5^ and by others in the setting of longitudinal data and mixed models.^18,19^ However, in the latter studies, the authors did not have to deal with the tricky issues posed by the use of a predicted latent variable as one of the regressors. Further research should strive to improve this.

Notice that, as shown in Taffé,^5^ the repeated measurements need not be from the reference standard, and the estimation method may be easily adapted to the setting where the repeated measurements come from the new method. This is a great asset of the proposed methodology, as sometimes it may turn out to be easier to perform many measurements by the new measurement method. Nevertheless, requiring repeated measurements by one of the two methods might discourage the applied researcher to use our methodology. However, this is necessary for statistical identification. Indeed, when the variance of the measurement errors of each measurement method is not constant or their ratio is unknown, which is usually the case in the biomedical field (the variance of measurement errors often increases as the latent trait increases), having only one measurement by each of the two measurement methods does not allow one to identify all the parameters of the model (2).^11^

The marginal probability of agreement has simply been estimated by the proportion of observations between the two tolerance limits and its confidence interval computed by the method of Agresti and Coull,^17^ although other methods such as that of Wilson may also have been used. We, also, have investigated the performance of our methodology for other distributions of the latent trait (Normal and Gamma, with skewness 1 and kurtosis 4.5, results not shown), and different parameter values for the heteroscedasticity. It still performed very well. This is the great asset of the empirical Bayes approach, which can accommodate virtually any distribution of the latent trait, given there are enough repeated measurements per individual.

We have seen that depending on how the tolerance limits have been defined (based or not on the value of the latent trait), the conditional agreement may be similar in shape to the percentage of agreement proposed by Taffé.^5^ As mentioned above, the former is based on pre-defined tolerance limits, whereas the latter depends on the width of the LoA and the amount of bias, and does not require the investigator to set tolerance limits. Which one should be preferred depends on the information available a priori to the investigator for setting the limits. We have illustrated that the definition of the tolerance limits may have an important leverage effect regarding the level of the conditional agreement calculated, whereas the percentage of the agreement depends solely on the variability and bias found in the data. It is recommended to compute both measures of agreement and thoroughly inspect the plots before deciding on the agreement.

Finally, it is important to emphasize that our modeling strategy rests on the assumption that the individual latent trait is constant within individuals, that is, $x_{ij}\equiv x_{i}$. This means that the repeated measurements should ideally be taken in sequence within a time interval where this assumption is sensible. It is theoretically possible to extend the methodology to other settings where the latent trait has a time trend.^4^ However, in that case, the simple and convenient decomposition of the bias into differential and proportional components is probably not sensible, and more sophisticated models should be developed.

In summary, we have extended the methodology proposed by Lin et al.^6^ and Stevens et al.,^7,8^ on the coverage probability/probability of agreement, by relaxing the strong parametric assumptions regarding the distribution of the latent trait and developing an inference method allowing us to compute pointwise and simultaneous CBs. The methodology requires repeated measurements by at least one of the two methods and can accommodate heteroscedastic measurement errors. It performs often very well even when one has only one measurement by one of the two methods and five to 10 repeated measurements from the other. This methodology will be made available in a future Stata package.

## Supplemental Material

sj-docx-1-smm-10.1177_09622802221137743 - Supplemental material for Use of clinical tolerance limits for assessing agreement Click here for additional data file. Supplemental material, sj-docx-1-smm-10.1177_09622802221137743 for Use of clinical tolerance limits for assessing agreement by Taffé Patrick in Statistical Methods in Medical Research
