# Supplementary material for: Use of clinical tolerance limits for assessing agreement
Source: Stat Methods Med Res. 2022 Nov 9;32(1):195–206. doi: 10.1177/09622802221137743 (PMC9814023; doi:10.1177/09622802221137743)
Supplement: sj-docx-1-smm-10.1177_09622802221137743 - Supplemental material for Use of clinical tolerance limits for assessing agreement [file sj-docx-1-smm-10.1177_09622802221137743.docx]

**Appendix : A worked example**

To illustrate the application of our methodology to real data, we re-used the data on diastolic blood pressure (DBP) from a previous study:^20^

**Supplementary Figure 1.** Scatter plot of the repeated DBP measurement pairs.

where the scatter plot presents the repeated DBP measurements made on each individual (10 repeated pairs, except for one patient where only 6 measurements were available) by the Microlife watchHomeBP (WBP) oscillometric blood pressure device (i.e. method 1) and the invasive arterial blood pressure (IBP) method based on an arterial indwelling catheter (i.e. method 2).

In this example, the clinical tolerance limits have been set as follows:

$$C_{L}(x_{i})=- 0.2 x_{i}$$

$$C_{U}(x_{i})=0.2 x_{i}$$

i.e. the tolerance limits are narrower for small values and larger for large values of the latent trait. As by definition $x_{i}$ is not observed, it is replaced by its best linear unbiased prediction (BLUP of x). Consequently, in the Tolerance limit plot the differences are allowed to vary around zero within a funnel-shaped band of width +- 20% of the BLUP of $x_{i}$ to declare agreement between the two paired measurements:

**Supplementary Figure 2.** Scatter plot of the differences y1-y2 (y1 = DBP WBP method and y2 = DBP IBP method) versus the BLUP of x with tolerance limits.

The conditional probability of agreement plot shows that the level of agreement is better for larger values of the latent trait:

**Supplementary Figure 3.** Conditional probability of agreement plot.

The pointwise 95% confidence band is useful for assessing the level of agreement at a specific value of the latent trait. However, when one is interested in assessing the agreement level at several values or even across an entire interval of the latent trait the simultaneous confidence band is required. Here, we see that the simultaneous band is, as expected, wider than the pointwise, to guarantee at least a 95% coverage rate for the simultaneous inference.
